# Circulating sphingosine-1-phosphate as a prognostic biomarker for community-acquired pneumonia

**DOI:** 10.1371/journal.pone.0216963

**Published:** 2019-05-15

**Authors:** Shih-Chang Hsu, Jer-Hwa Chang, Yuan-Pin Hsu, Kuan-Jen Bai, Shau-Ku Huang, Chin-Wang Hsu

**Affiliations:** 1 Emergency Department, Department of Emergency and Critical Medicine, Wan Fang Hospital, Taipei Medical University, Taipei, Taiwan; 2 Department of Emergency Medicine, School of Medicine, College of Medicine, Taipei Medical University, Taipei, Taiwan; 3 Division of Pulmonary Medicine, Department of Internal Medicine, School of Medicine, College of Medicine, Taipei Medical University, Taipei, Taiwan; 4 Division of Pulmonary Medicine, Department of Internal Medicine, Wan Fang Hospital, Taipei Medical University, Taipei, Taiwan; 5 Graduate Institute of Clinical Medicine, College of Medicine, Taipei Medical University, Taipei, Taiwan; 6 National Institute of Environmental Health Sciences, National Health Research Institutes, Miaoli County, Taiwan; 7 Lou-Hu Hospital, Shen-Zhen University, Shen-Zhen, China; 8 Research Center for Environmental Medicine, Kaohsiung Medical University, Kaohsiung, Taiwan; 9 Johns Hopkins Asthma and Allergy Center, Johns Hopkins University School of Medicine, Baltimore, United States of America; National Yang-Ming University, TAIWAN

## Abstract

Early determination of the severity of Community-Acquired Pneumonia (CAP) is essential for better disease prognosis. Current predictors are suboptimal, and their clinical utility remains to be defined, highlighting the need for developing biomarkers with efficacious prognostic value. Sphingosine-1-phosphate (S1P) is a bioactive sphingolipid with a documented regulatory role in immune defense and maintenance of endothelial barrier integrity. For early diagnose of CAP and recognition of severe CAP patients, we conduct this pilot study to access the potential utility of the circulating S1P in an Emergency department setting. In the prospective study, plasma S1P levels were quantified in healthy controls and patients with CAP. Also, their discriminating power was assessed by receiver operating characteristic analysis. The association between S1P levels and disease severity indices was assessed by Spearman correlation and logistic regression tests. Patients with CAP had significantly higher plasma S1P levels than healthy individuals (CAP: 27.54 ng/ml, IQR = 14.37–49.99 ng/ml; Controls: 10.58 ng/ml, IQR = 4.781–18.91 ng/ml; p < 0.0001). S1P levels were inversely correlated with disease severity in patients with CAP. Based on multivariate logistic regression analysis, the plasma S1P concentrations showed significant predicting power for mortality (OR: 0.909; CI: 0.801–0.985; p < 0.05), intensive care unit admission (OR: 0.89; CI: 0.812–0.953; p < 0.005) and long hospital stay (OR: 0.978; CI: 0.961–0.992; p < 0.005). Interestingly, significantly elevated levels of S1P were noted in patients who received methylprednisolone treatment during hospitalization. These results suggest that S1P may be associated with the pathogenesis of CAP and may have prognostic utility in CAP and its therapy, especially in the Emergency Department setting.

## Introduction

Lower respiratory tract infections are the most frequent infectious cause of death worldwide [1] and impose a considerable burden on healthcare resources. Despite the advancement in treatment and diagnosis, the inpatient mortality rate of community-acquired pneumonia (CAP) is 5.7% to 14.0% [2,3]. Early stratifying the severity of CAP is thus very important, especially in an acute emergency setting. Moreover, delayed intensive care unit (ICU) admission is associated with increased CAP mortality[4]. The pneumonia severity index (PSI) [5] and CURB-65 [3] are two well-known clinical CAP specific scores for identifying low-risk individuals who are candidates for outpatient care, but these scores do not perform well in predicting the need for ICU admission [6].

C-reactive protein (CRP) and procalcitonin (PCT) have been widely used in pneumonia management [7]. CRP is a well-established biomarker of inflammation but has been considered as a non-specific marker in the pneumonia diagnosis [8]. However, some studies have shown that it might have some values in defining pneumonia severity [9,10]. PCT, another inflammatory biomarker, has been extensively evaluated as a marker for bacterial infectious disease severity and progression [11,12]. For CAP, however, the prognostic accuracy of PCT is not optimal. In a serial measurement, increased PCT was significantly related to increasing severity of CAP; however, a single measurement of PCT on admission is not adequate for prognostic assessment [13]. Moreover, several meta-analyses have suggested that both biomarkers perform no better than the CAP-specific scores in prognostic prediction [14,15] and that these biomarkers are suggested to have better value in monitoring the treatment response than as a single point-of-care prognostic assessment tool [16]. Therefore, developing new biomarkers for predicting CAP severity in the early disease phase would be needed.

Sphingosine-1-phosphate (S1P) is a bioactive sphingolipid and has both extracellular and intracellular effects on mammalian cells [17–19]. S1P is synthesized by two sphingosine kinases (SphK1 and SphK 2) and degraded by S1P lyase (S1PL) [17]. S1P is a ligand for five G protein-coupled receptors, S1P receptors1–5 [17,18], and also acts as an intracellular second messenger [20,21]. S1P is involved in many physiological processes, including immune responses and endothelial barrier integrity [22–25]. Also, S1P plays a crucial role in protecting the lungs from the pulmonary leak and lung injury [26–29]. Previous research also suggests that S1P signaling through S1P receptor 1 (S1PR1) is vital for endothelial barrier function [30]. Because of the involvement in lung injury and endothelial barrier function, S1P could be a potential biomarker of pneumonia. In the present study, we evaluated the diagnostic value of S1P in patients who presented at the Emergency Department (ED) with CAP. The prognostic value of S1P on short-term outcomes, such as the length of hospital stay, ICU admission, and hospital mortality were also investigated.

## Materials and methods

This observational, prospective, single-center, case-control study was approved by Association of Taipei Medical University Joint Institutional Review Board (TMU-JIRB NO: N201602089), and all experiments in this research were performed in accordance with the relevant guidelines and regulations. The study is registered on ClinicalTrials.gov (NCT03473119). The study objects were enrolled in Wan Fang Medical Center (Taipei, Taiwan) between October 2016 and April 2018.

### Study population and clinical variables

The study group consisted of patients with a diagnosis of CAP who presented to the ED. The control group comprised healthy adults who accompanied the patients were also recruited from the ED. All recruited individuals were provided with written informed consent before enrollment. The inclusion criteria were: age ≧ 20 years and suspected diagnosis of CAP as defined by the Infectious Disease Society of America (IDSA)/ American Thoracic Society (ATS) Consensus Guideline [31]. Briefly, pneumonia was defined as a new pulmonary infiltrate on the chest radiograph with symptoms and signs of lower respiratory tract infection.

The exclusion criteria were: pneumonia in the previous 30 days, active tuberculosis, suspected aspiration pneumonia (The patient who had a witnessed aspiration (choking) and the aspiration is shortly followed by coughing, shortness of breath, or tachypnea.), immune-deficiency (due to HIV infection, prior transplantation, immunosuppressive therapy or neoplasm) and pregnancy. Upon admission to the ED, the patient’s demographic and clinical histories were recorded. The clinical (blood pressure, heart rate, respiratory rate, and body temperature) and laboratory parameters (Complete Blood Count with differential, CRP, renal function, and electrolytes) were then collected. To evaluate the diagnostic and prognostic abilities of S1P, the initial blood sample for S1P measurement was collected before any treatment. To assess if the S1P level would return to baseline after successful treatment, the blood sample for S1P measurement was collected again one day before discharge.

The PSI and CURB-65 were calculated according to the international criteria. Based on the PSI and CURB65, the severity of pneumonia was then classified into low (PSI: ≦ 90; CURB-65: 0–1), moderate (PSI: 91–130; CURB-65: 2) and high (PSI: > 130; CURB-65: 3–5). The scores of PSI and the CURB-65 are correlated with each other and if use them to evaluate the same population usually will give rise to comparable results. In this study, for risk stratification, we mainly focus on PSI. Fifty patients with pneumonia received corticosteroid therapy during the hospitalization. The reasons for giving corticosteroid as following: Septic shock (n = 25), Acute exacerbations of chronic obstructive pulmonary disease (n = 13) and Corticosteroid adjuvant therapy (n = 12). The final diagnosis was provided by the follow-up or the admitting pulmonologists.

### Measurement of sphingosine-1-phosphate

The collected blood samples were placed in tubes containing EDTA, immediately centrifuged at 2500xg for 10 minutes and the upper remaining plasma parts were collected into Eppendorf microcentrifuge tubes. The samples were stored frozen at -80°C until the day of S1P analysis. The S1P levels in the plasma samples were measured by enzyme-linked immunosorbent assay (ELISA) kit (MyBiosource).

### Statistical analysis

Statistical analysis of the data obtained in the study was made using R 3.2.4 software (R Foundation for Statistical Computing, Vienna, Austria). Continuous variables were expressed as means and standard deviation (SD) or medians and the interquartile range. The categorical variables were expressed as counts or percentages. The categorized data was assessed by using Fisher’s direct exact test. The Mann-Whitney U-test was used for continues variable that did not follow a parametric distribution. The degree of association between variables was measured by the Spearman rank correlation test. Using receiver operating characteristic (ROC) curve analysis, the area under the curve (AUC) and the cut-off values (determined by Youden indexes) were calculated. Comparing ROC curves was done using the empirical (nonparametric) methods as described in Ref. [32].

A univariate analysis screening method to select covariates for multiple logistic regression was used in the study. Univariate analysis was initially used on all variables. Variables that were significant in univariate analysis were included in a multiple logistic regression analysis to identify independent predictors. Univariate logistic regression analysis was performed to predict overall hospital stay > 10 days, ICU admission and hospital mortality. Since CRP was not significant in univariate analysis, it was not included in the multivariate analysis. For the hospital mortality and ICU admission, S1P, PSI and CURB 65 were included in the multivariate analyses. In term of hospital stay > 10 days, S1P, CRP, PSI and CURB 65 were included in the multivariate analyses. Since the information of age, sex, and comorbidity were used in PSI calculation, we did not include them into the models. Statistical tests were two-sided, and p values less than 0.05 were considered statistically significant.

## Results

### Characteristics of the study population

Initially, 160 patients were assessed and a total of 23 patients were excluded due to the following: 4 patients with pneumonia in the previous 30 days, 3 patients with aspiration pneumonia, 3 patients with tuberculosis pulmonary infection, 10 patients with final non-pneumonia diagnosis (Influenza A or B, Acute myocardial infarction, acute heart failure, septic shock with liver abscess, septic shock with acute cholangitis, etc) and 3 patients with uncertain diagnosis, and 137 patients were finally included in the study. Also, 78 healthy volunteers were also recruited. Second blood samples (one day before discharge) were available for 71 patients (S1 Fig). Demographic and clinical characteristics are summarized in Table 1. A total of 215 individuals were included in the analysis, and the age significantly differed between the controls and CAP patients. Of the 137 pneumonia patients, 123 were admitted, 21 required ICU-level cares, and eight eventually died. The CAP patients were also assigned to different risk levels [Low: 43(31.39%), Moderate: 64(46.71%) and High: 30(21.90%)] according to the PSI score. Of those admitted patients, the median length of hospital stay was 9 (IQR: 7–13) days.

**Table 1:** Characteristics of control and study groups.

Variables	Controls (n = 78)	CAP patients (n = 137)	p-value
Age (years; Mean ± SD)	55.83 ± 18.35	73.41 ± 16.83	< 0.01
Male/Female n(%)	42/36(53.85%)	83/54 (60.58%)	0.389
Admission n(%)	NA	123 (89.78%)	
ICU admission n(%)	NA	21 (17.07%)	
Length of stay (Days; Median, IQR)	NA	9 (7–13)	
Hospital mortality n(%)	NA	8 (6.50%)	
PSI			
≦90	NA	43 (31.39%)	
91–130	NA	64 (46.71%)	
>130	NA	30 (21.90%)	
CURB-65			
0–1	NA	73 (53.28%)	
2	NA	36 (26.28%)	
3–5	NA	28 (20.44%)	
Comorbidities			
Hypertension	29 (37.18%)	65 (47.45%)	0.155
Diabetes mellitus	17 (21.79%)	36 (26.28%)	0.513

### Concentrations of plasma S1P upon ED admission

**Fig 1:**
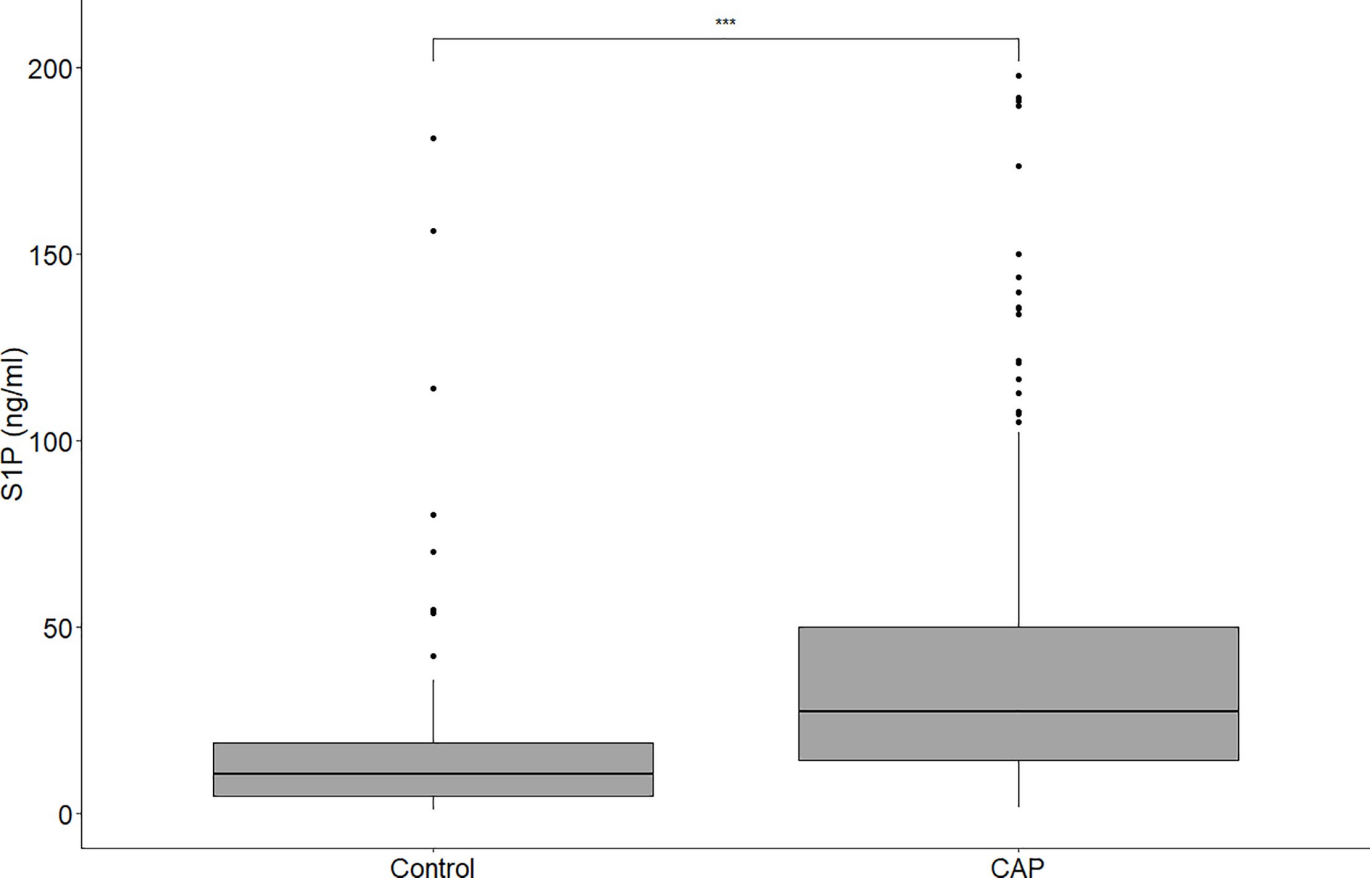
The distribution of plasma S1P levels in controls and patients with CAP (*** p <0.0001).

S1P concentrations ranged from 1.11 ng/ml to 200.00 ng/ml. Patients with CAP had significantly higher S1P values as compared to those in control objects (controls: 10.58 ng/ml, IQR = 4.781–18.91 ng/ml; patient: 27.54 ng/ml, IQR = 14.37–49.99 ng/ml; p < 0.0001; Fig 1). The area under the ROC curve for S1P level was 0.744(95% CI: 0.674–0.813) with sensitivity of 69.2% and specificity of 70.8% at 15.56 ng/ml (S2 Fig). In the univariate analysis, S1P concentration was predictive of CAP with odds ratio of 1.021 (95% CI: 1.010–1.035; p < 0.0005). The results of the full panel of the univariate and multivariate logistic regression analysis were provided in S1 Table.

### Prognostic analysis

**Fig 2:**
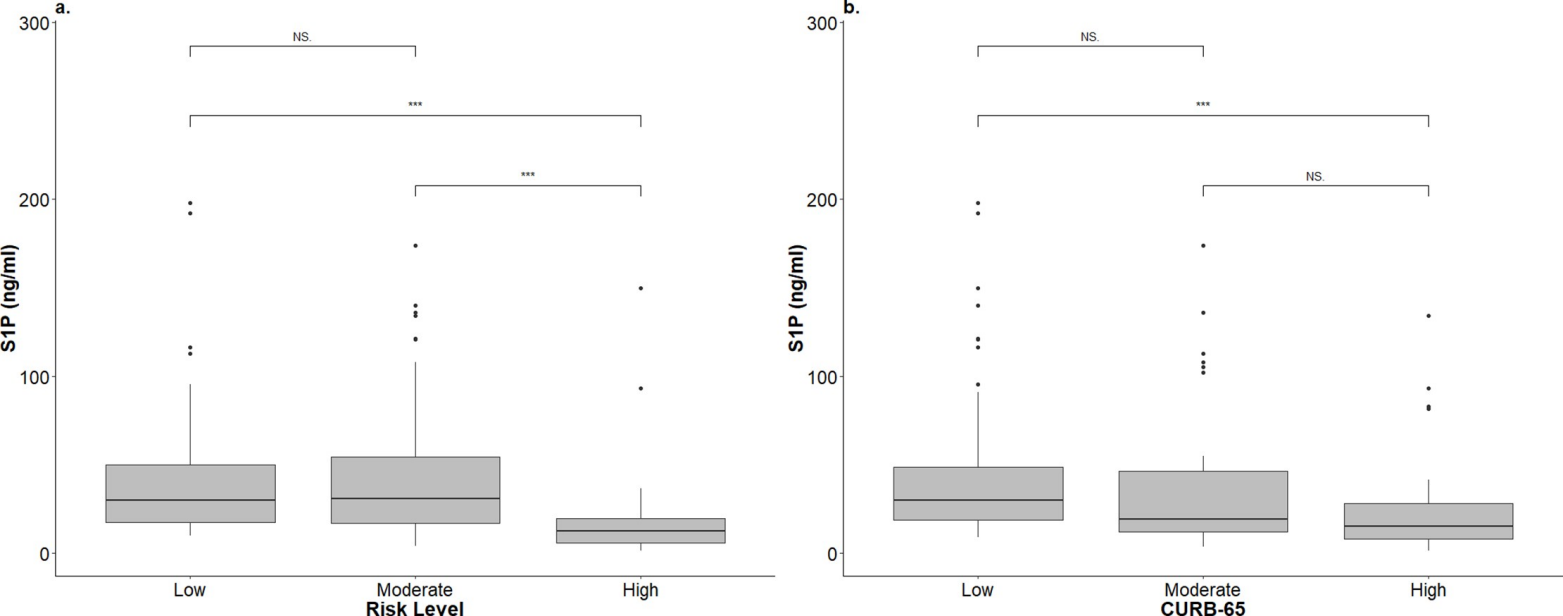
The distribution of plasma S1P levels in different disease severity groups. (a) plasma S1P level distribution in different mortality risk (Pneumonia Severity Index) group. (b) plasma S1P level distribution in different CURB-65 classes. (NS. Non-significant, ** p<0.001, *** p <0.0001).

To determine the correlation between S1P values and the severity of CAP, PSI, CURB-65, and length of hospital stay (LOS) were used as pneumonia severity indices. For comparison, the common biomarkers used in infection were included for analysis and correlation between CRP and pneumonia severity were also analyzed (S2 Table). There were significant correlations between the level of S1P and PSI (rho = -0.378, p < 0.0001), CURB-65 (rho = -0.346, p < 0.0001), LOS (rho = -0.509, p < 0.00001). In contrast, significant correlation was only noted between CRP and LOS (rho = 0.23, p < 0.015). Further, when the levels of S1P were stratified by different risk groups based on the PSI and CURB-65 values, significantly lower levels of S1P were seen in the high-risk patient group than those noted in the low- and moderate-risk groups (Fig 2A). Regarding the CURB-65 score, patients in the low-risk group had a significantly higher level of S1P than patients in the high-risk group (Fig 2B)

**Fig 3:**
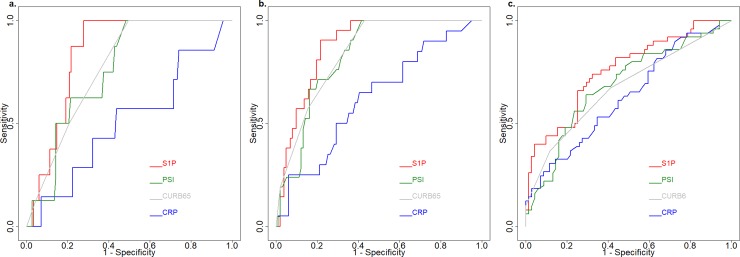
Receiver operating characteristic (ROC) curves for the prediction of different study outcomes: (a) Hospital mortality. S1P: AUC = 0.843 (95% CI: 0.764–0.922); PSI: AUC = 0.761 (95% CI: 0.636–0.885); CURB-65: AUC = 0.774 (95% CI: 0.676–0.889); CRP: AUC = 0.501 (95% CI: 0.272–0.7472). (b) ICU admission. S1P: AUC = 0.878 (95% CI: 0.816–0.940); PSI: AUC = 0.829 (95% CI: 0.752–0.904); CURB-65: AUC = 0.828 (95% CI: 0.771–0.909) CRP: AUC = 0.623 (95% CI: 0.489–0.756). (c) Hospital stay longer than ten days. S1P: AUC = 0.756 (CI: 0.668–0.843); PSI: AUC = 0.686 (95% CI: 0.587–0.784); CURB-65: AUC = 0.672 (95% CI: 0.578–0.766); CRP: AUC = 0.628 (CI: 0.526–0.730).

Next, in the ROC analysis, S1P showed the highest AUC value for the prediction of hospital mortality, ICU admission, and the hospital stay longer than ten days (Fig 3). By comparing with CRP, S1P had significantly higher AUC value for hospital mortality (p < 0.005), ICU admission (p < 0.0005), and the hospital stay longer than ten days (p < 0.05). There were no statistically significant differences between PSI, CURB-65, and S1P in predicting hospital mortality, ICU admission or long hospital stay. Also, both PSI and CURB-65 had significantly higher AUC values for ICU admission (PSI: p < 0.001; CURB_65: p <0.005) than that of CRP. In the univariate analysis, both S1P level and PSI score were associated with hospital mortality and ICU admission and all three predictors were associated with hospital stay longer than ten days (Table 2). Further, in the multivariate logistic regression model, only the S1P level was identified as an independent predictor for all three disease severity indices (Table 2).

**Table 2:** Prognostic effect of S1P level, CRP level, PSI, and CURB65 upon emergency department admission for hospital mortality, ICU admission, and hospital stay longer than ten days in univariate and multivariate logistic regression analysis.

**Univariate Analysis**						
	**Hospital Mortality**		**ICU Admission**		**LOS > 10 days**	
Variable	OR	p-value	OR	p-value	OR	p-value
S1P	0.883	<0.020*	0.86	<0.0005*	0.971	<0.001*
	CI: 0.782–0.961		CI: 0.786–0.920		CI: 0952–0.986	
CRP	0.995	0.943	1.057	0.116	1.086	<0.01*
	CI: 0.864–1.110		CI: 0.984–1.133		CI: 1.023–1.159	
PSI	1.024	< 0.05*	1.039	<0.0005*	1.021	<0.005*
	CI: 1.002–1.048		CI: 1.020–1.062		CI: 1.008–1.036	
CURB65	2.594	< 0.05*	4.371	<0.0005*	2.208	<0.005*
	CI: 1.262–5.789		CI: 2.431–8.811		CI: 1.446–3.509	
	**Multivariate Analysis**					
	**Hospital Mortality**		**ICU Admission**		**LOS > 10 days**	
Variable	OR	p-value	OR	p-value	OR	p-value
S1P	0.909	< 0.05*	0.89	< 0.005*	0.978	<0.005*
	CI: 0.801–0.985		CI: 0.812–0.953		CI: 0.961–0.992	
CRP	-	-	-	-	1.076	< .05*
	-		-		CI: 1.008–1.159	
PSI	1.003	0.829	1.005	0.681	1.008	0.384
	CI: 0.971–1.033		CI: 0.908–1.033		CI: 0.991–1.027	
CURB65	1.624	0.334	3.098	<0.05*	1.512	0.195

* Statistical significance

### Levels of Plasma S1P one day before discharge

**Fig 4:**
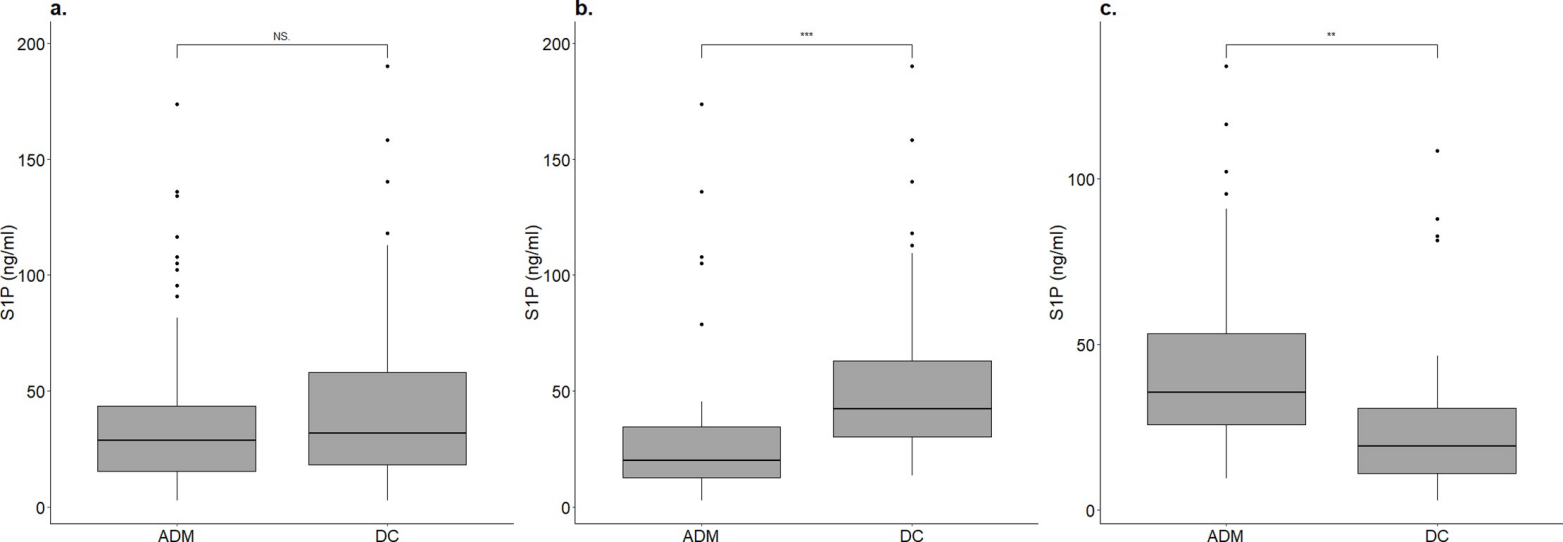
The distribution of plasma S1P levels in patients with CAP upon emergency department admission (ADM) and one day before discharge (DC). (a) Total (n = 71). (b) With corticosteroid treatment during the hospitalization (n = 41). (c) Without corticosteroid treatment during the hospitalization (n = 30). (NS. Non-significant, ** p <0.001, *** p <0.0001).

The S1P level in blood samples drawn one day before discharge was also measured. There was no significant difference between the S1P level at admission and one day before discharge (Admission: 28.72 ng/ml, IQR = 15.51–43.40 ng/ml; Discharge:31.93 ng/ml, IQR = 18.11–58.072 ng/ml; p = 0.23; Fig 4A). The patients were further separated into two groups based on corticosteroid usage during hospitalization. In this study population, 50 patients received corticosteroid therapy, and blood samples were available for 41 patients. For 73 patients without corticosteroid treatment, 30 blood samples were available. Results showed that the level of S1P was significantly elevated in patients receiving corticosteroid treatment during hospitalization (Admission: 20.17 ng/ml, IQR = 12.67–34.72 ng/ml; Discharge: 42.23 ng/ml, IQR = 30.29–62.93 ng/ml; p < 0.0001; Fig 4B). However, without corticosteroid therapy, the levels of S1P at one day before discharge were significantly decreased (Admission: 35.41 ng/ml, IQR = 25.78–53.14 ng/ml; Discharge: 19.17 ng/ml, IQR = 11.00–30.66 ng/ml; p > 0.001; Fig 4C). Further, the patients at admission in the non-corticosteroid treatment group had a significantly higher levels of plasma S1P than those noted in the corticosteroid treatment group (p < 0.01). Since the baselines of S1P level in patients with or without receiving steroid were different, we further divided the patients into two groups: high S1P (>25 ng/ml) and low S1P (≦25 ng/ml). After the stratification, the analyses of the patients with corticosteroid treatment showed similar results (S3A, S3B and S3D Fig). However, in the low S1P group, among patients without corticosteroid treatment, there was no statistically significant difference between the S1P level upon admission and one day before discharge (S3C Fig).

## Discussion

The results of our prospective case-control study indicated that plasma S1P levels were significantly increased in patients with CAP, compared to those of the healthy controls. The level of circulating S1P at the time of ER admittance was found to predict mortality, ICU admission and the hospital stay longer than ten days in patients with pneumonia. Moreover, we also showed that the circulating level of S1P could be associated with corticosteroid usage. To our knowledge, this is the first study to investigate the S1P as a potential biomarker in patients with pneumonia and provide evidence for its association with corticosteroid adjuvant therapy.

CAP is a leading cause of sepsis, and the early estimation of disease severity is essential to reduce pneumonia-related morbidity and mortality [4,33]. In this study, we first demonstrated that the patients with CAP had an elevated level of plasma S1P by comparing with the healthy controls. ROC analysis also suggested that S1P could potentially be a sensitive and specific novel biomarker aiding the diagnosis of CAP in an acute ED setting. Although in this dataset, the age of the controls significantly differed from the age of the patients, the age and gender did not influence the S1P level [34,35]. It was also noted that in the same ER setting, while limited in sample size, no significant elevation of circulating S1P was seen in patients with chronic obstructive pulmonary disease (COPD; N = 21) during an exacerbation, as compared with that in the healthy control group (unpublished observation). In contrast, an increasing trend of the S1P level was noted in a panel of COPD patients with pneumonia (N = 30), suggesting selective upregulation of S1P in pneumonia cases.

While the PSI is a common known CAP specific score, it is infrequently used in routine clinical practice, especially in an emergency setting, mainly due to a high number of required variables [36]. Also, PSI performs less well in predicting the need for ICU admission in patients with CAP [6]. Several biomarkers, such as PCT, pro-adrenomedullin, atrial natriuretic peptide (ANP), copeptin, cortisol, and CRP, have been evaluated for prediction of prognosis in CAP. However, none of those biomarkers performed significantly better than the CAP-specific score [14]. Thus, biomarkers that can detect patients with poor prognosis in the early phase of the disease period would help physicians to modify the initial management of the patients with CAP and improve the disease outcomes. Further, the S1P levels, but not CRP, were found to be inversely correlated with PSI score, CURB-65 score and hospital length of stay (LOS) in patients with CAP. ROC analysis suggested that S1P level had the highest AUC values in the prediction of mortality, ICU admission, and hospital stay longer than ten days in patients with pneumonia. When the patients were stratified by different risk levels based on PSI or CURB-65, the patients in the high-risk group had significantly lower plasma S1P levels. Furthermore, in the multivariate logistic regression model, S1P was shown to be the only significant predictor of mortality, ICU admission and hospital stay longer than ten days. Based on these results, plasma S1P might not be an ideal biomarker for pneumonia diagnosis, because the patient with severe pneumonia would have a lower level of plasma S1P. Therefore, for the diagnosis, S1P plus other biomarkers such as CRP or PCT to create a multi-biomarker tool would be needed. Nonetheless, plasma S1P could be a promising biomarker for predicting pneumonia prognosis in the early disease phase, especially in an emergency department setting.

In the context of infection, S1P has been shown to influence several types of cells involved in immune responses, including neutrophil activation and recruitment [37,38] as well as egress of lymphocytes into the circulation [22,39]. In addition, S1P is suggested to be involved in B-cell migration [40,41]. Several studies have suggested that S1P can enhance pulmonary endothelial cell barrier function [26,27,29], suggesting that higher S1P levels could be potentially beneficial. Hence, the patients, who are unable to produce sufficient S1P, might have a poor prognosis. However, in most of the studies, CAP was not considered as a disease model, and those results were based on cell lines and mouse models. Therefore, further studies focusing on the role of S1P in the pathophysiology of pneumonia is needed.

Although a recent meta-analysis study shows that corticosteroid adjuvant therapy in patients with severe CAP could reduce the rate of hospital mortality, the length of ICU stay, and the length of hospital stay, corticosteroid adjuvant therapy for CAP is still controversial [42–44]. There has been no standard criteria or biomarker for initiating corticosteroid adjuvant therapy. Also recently, the long-standing dogma of cytokine repression by the glucocorticoid was challenged. Vettorazzi et al. proposed a new mechanism of glucocorticoid action through the activation of sphingosine kinase 1 (SphK1), and hence the increase of circulating S1P levels, by glucocorticoids, which was suggested to be essential for the inhibition of pulmonary inflammation [45]. Furthermore, in a mouse model, the macrophage population was shown to be responsible for the elevated level of S1P in plasma. Interestingly, our observational study also showed the significantly elevated S1P level in patients who were treated with methylprednisolone during hospitalization. Besides, S1P level did not rise in a small number of patients with pneumonia who did not receive methylprednisolone throughout the hospitalization.

Based on the above evidence, we hypothesized that the S1P/S1PR1-signaling pathway might play a vital role in the pathobiology of pneumonia. In terms of pneumonia, the two important functions of S1P are an enhancement of pulmonary endothelial cell barrier function and inhibition of pulmonary inflammation. Hence, the patients, who are unable to produce sufficient S1P, might have a poor prognosis. The corticosteroid adjuvant therapy may only be beneficial for patients with CAP who were unable to produce a sufficient amount of S1P. Therefore, S1P could be a potential biomarker candidate for deciding the use of corticosteroids adjuvant therapy.

Several limitations of this study are noted. This was conducted at the ED of a single center and involved a relatively small number of patients with CAP. We were unable to obtain the second blood sample of the patient who had expired during hospitalization, and the low percentage of study patients has a blood test of S1P one day before discharge are both important limitations. Also, as this is an observational study, the corticosteroid treatment cannot be controlled. Since the effect of corticosteroid is an accidental finding, the study is not specifically designed for it. This makes further analysis of corticosteroid effects not possible. To confirm the effect of corticosteroid on S1P, the additional studies are needed. Finally, because the mean age of the CAP patients is relatively older in our study (mean age = 73), whether the findings of this study could be applied to a younger population should be further investigated. To propose S1P as a routine CAP biomarker in the Emergency Department setting, expanded sample size and multi-center studies will be needed to further validate our findings. Besides, the serial evaluation of S1P during hospitalization and then to elucidate the role of S1P in CAP diagnosis and predicting outcome should be further investigated.

## Conclusions

Plasma S1P levels were significantly elevated and inversely correlated with disease severity in patients with CAP. The plasma S1P level was also noted as a good predictor of mortality, ICU admission and hospital stay longer than ten days. S1P appeared to be a potential prognostic biomarker for the initial screening of patients with CAP in the Emergency Department. Our observation of higher plasma S1P levels seen in patients who were treated with corticosteroid suggested that S1P could be a potential biomarker candidate for guiding the usage of corticosteroids adjuvant therapy.

## Supporting information

S1 Fig Study scheme. (TIFF)

S2 Fig Receiver operating characteristic (ROC) curve for the CAP diagnosis of plasma S1P levels on emergency department admission. (TIFF)

S3 Fig The distribution of stratified plasma S1P levels in patients with CAP upon emergency department admission (ADM) and one day before discharge (DC). (a) Low S1P level with corticosteroid treatmemt; n = 23 (Admission: 12.70 ng/ml, IQR = 7.27–17.99 ng/ml; Discharge: 32.32 ng/ml, IQR = 21.73–50.78 ng/ml). (b) High S1P level with corticosteroid treatmemt; n = 18 (Admission: 38.85 ng/ml, IQR = 28.96–70.46 ng/ml; Discharge:61.76 ng/ml, IQR = 42.78–98.64 ng/ml). (c) Low S1P level without corticosteroid treatmemt; n = 7 (Admission: 15.60 ng/ml, IQR = 13.46–17.61 ng/ml; Discharge:19.18 ng/ml, IQR = 11.52–32.44 ng/ml). (d) High S1P level without corticosteroid treatmemt; n = 23 (Admission: 41.47 ng/ml, IQR = 33.48–72.26 ng/ml; Discharge:14.39 ng/ml, IQR = 11.08–22.17 ng/ml). (NS. Non-significant, * p <0.05, *** p <0.0001).
(TIFF)

S1 Table Diagnostic effect of S1P level, CRP level, upon emergency department admission for CAP in univariate and multivariate logistic regression analysis. (DOCX)

S2 Table Correlation of CRP and S1P with different pneumonia disease severity indices. (DOCX)

S1 Dataset Dataset used in the present study. (XLSX)
