# Supplementary figures and images for: Circulating sphingosine-1-phosphate as a prognostic biomarker for community-acquired pneumonia
Source: PLoS One. 2019 May 15;14(5):e0216963. doi: 10.1371/journal.pone.0216963 (PMC6519827; doi:10.1371/journal.pone.0216963)

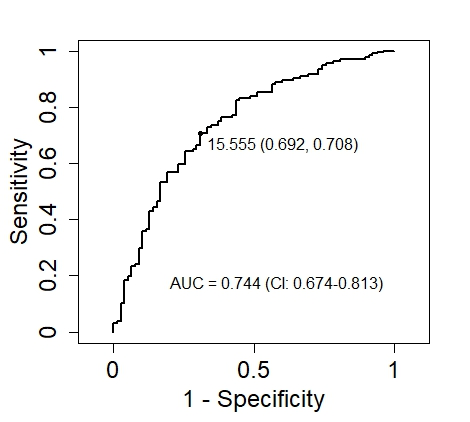

Supplement: S2 Fig — (TIFF) [file pone.0216963.s002.tiff]

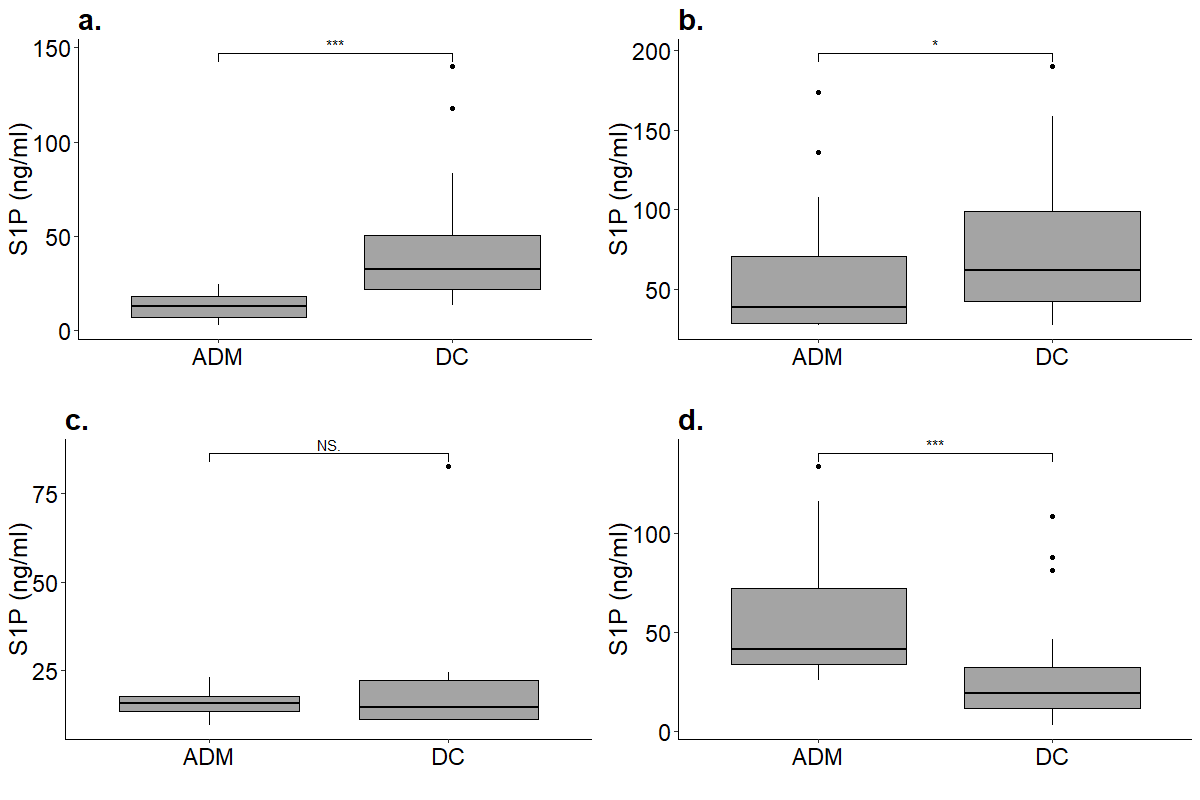

Supplement: S3 Fig — (a) Low S1P level with corticosteroid treatmemt; n = 23 (Admission: 12.70 ng/ml, IQR = 7.27–17.99 ng/ml; Discharge: 32.32 ng/ml, IQR = 21.73–50.78 ng/ml). (b) High S1P level with corticosteroid treatmemt; n = 18 (Admission: 38.85 ng/ml, IQR = 28.96–70.46 ng/ml; Discharge:61.76 ng/ml, IQR = 42.78–98.64 ng/ml). (c) Low S1P level without corticosteroid treatmemt; n = 7 (Admission: 15.60 ng/ml, IQR = 13.46–17.61 ng/ml; Discharge:19.18 ng/ml, IQR = 11.52–32.44 ng/ml). (d) High S1P level without corticosteroid treatmemt; n = 23 (Admission: 41.47 ng/ml, IQR = 33.48–72.26 ng/ml; Discharge:14.39 ng/ml, IQR = 11.08–22.17 ng/ml). (NS. Non-significant, * p <0.05, *** p <0.0001). (TIFF) [file pone.0216963.s003.tiff]
