# Supplementary material for: Circulating sphingosine-1-phosphate as a prognostic biomarker for community-acquired pneumonia
Source: PLoS One. 2019 May 15;14(5):e0216963. doi: 10.1371/journal.pone.0216963 (PMC6519827; doi:10.1371/journal.pone.0216963)
Supplement: S1 Table — (DOCX) [file pone.0216963.s004.docx]

**S1 Table.** Diagnostic effect of S1P level, CRP level, upon emergency department admission for CAP in univariate and multivariate logistic regression analysis.

| **CAP** |  | **Univariate** | |  | **Multivariate** | |
| --- | --- | --- | --- | --- | --- | --- |
|  | Variable | OR | p-value | Variable | OR | p-value |
|  | **S1P** | 1.021 | <0.0005* | **S1P** | 1.119 | <1x10^-7^* |
|  |  | CI: 1.010-1.035 |  |  | CI: 1.070-1.183 |  |
|  |  |  |  |  |  |  |
|  | **CRP** | 1.738 | < 5x10^-6^* | **CRP** | 1.655 | <2.5x10^-5^* |
|  |  | CI: 1.417-2.256 |  |  | CI: 1.353-2.165 |  |

* Statistical significance
