# Supplementary material for: Circulating sphingosine-1-phosphate as a prognostic biomarker for community-acquired pneumonia
Source: PLoS One. 2019 May 15;14(5):e0216963. doi: 10.1371/journal.pone.0216963 (PMC6519827; doi:10.1371/journal.pone.0216963)
Supplement: S2 Table — (DOCX) [file pone.0216963.s005.docx]

**S2 Table.** Correlation of CRP and S1P with different pneumonia disease severity indices.

| **Variables** | **PSI** | | **CURB-65** | | **LOS** | |
| --- | --- | --- | --- | --- | --- | --- |
|  | Spearman rho | p value | Spearman rho | p value | Spearman rho | p value |
| S1P | -0.378 | <0.0001* | -0.356 | <0.0001* | -0.509 | <0.00001* |
| CRP | 0.091 | 0.327 | -0.005 | 0.954 | 0.23 | 0.015* |

* Statistical significance
